# Time-evolving sea-surface warming patterns modulate the climate change response of subtropical precipitation over land

**DOI:** 10.1073/pnas.1911015117

**Published:** 2020-02-18

**Authors:** Giuseppe Zappa, Paulo Ceppi, Theodore G. Shepherd

**Affiliations:** ^a^Department of Meteorology, University of Reading, Reading RG6 6BB, United Kingdom;; ^b^Istituto di Scienze dell’Atmosfera e del Clima, Consiglio Nazionale delle Ricerche, Bologna 40129, Italy;; ^c^Grantham Institute for Climate Change and the Environment, Imperial College London, London SW7 2AZ, United Kingdom

**Keywords:** climate change, hydrological cycle, Mediterranean climates, CMIP5

## Abstract

Greenhouse gas (GHG) emissions affect precipitation worldwide. The response is commonly described by two timescales linked to different processes: a rapid adjustment to radiative forcing, followed by a slower response to surface warming. However, additional timescales exist in the surface-warming response, tied to the time evolution of the sea-surface-temperature (SST) response. Here, we show that in climate model projections, the rapid adjustment and surface mean warming are insufficient to explain the time evolution of the hydro-climate response in three key Mediterranean-like areas—namely, California, Chile, and the Mediterranean. The time evolution of those responses critically depends on distinct shifts in the regional atmospheric circulation associated with the existence of distinct fast and slow SST warming patterns. As a result, Mediterranean and Chilean drying are in quasiequilibrium with GHG concentrations, meaning that the drying will not continue after GHG concentrations are stabilized, whereas California wetting will largely emerge only after GHG concentrations are stabilized. The rapid adjustment contributes to a reduction in precipitation, but has a limited impact on the balance between precipitation and evaporation. In these Mediterranean-like regions, future hydro-climate–related impacts will be substantially modulated by the time evolution of the pattern of SST warming that is realized in the real world.

## Significance

One of the most impactful aspects of climate change is the potential change in water availability. Large populations live in Mediterranean-like regions—so-called because they receive most of their precipitation in winter and experience dry, hot summers—which are highly vulnerable to water stress. It is generally assumed that changes in water availability are proportional to global warming. In this study, we show that this is not the case for Mediterranean-like regions, because of the strong influence of changing patterns of atmospheric circulation induced by patterns of sea-surface-temperature changes. In the Mediterranean itself and in Chile, the projected drying is substantially accelerated relative to global warming, whereas in California, the projected moistening is substantially delayed.

Changes in regional precipitation and hydro-climate are among the most impact-relevant aspects of climate change. Particular concern regards the projected drying of subtropical and Mediterranean-like land regions, where water resources are already limited, susceptible to large year-to-year variability, and under the pressure of increasing population (1). It is often assumed that the amplitude of regional climate responses scales approximately linearly with global-mean warming (2, 3), but such a behavior is not guaranteed on physical grounds (4) and indeed not always found in model projections (5, 6). Increasing confidence in the time evolution of regional precipitation changes requires understanding the driving physical processes and the timescales on which they act.

It is a standard approach to consider the climate response as the sum of a rapid adjustment proportional to the radiative forcing and a slower response proportional to surface warming (7). The presence of such distinct timescales is evident in coupled experiments forced by abrupt increases in greenhouse gas (GHG) concentrations, so that the climate impacts of radiative forcing and warming are well separated. For hydrological changes, this framework has successfully explained the time evolution of the global-mean precipitation response to GHG increases, which drops (rapidly) due to changes in the atmospheric radiative absorption, before increasing (slowly) with global surface warming (7–9).

This two-timescale decomposition has been recently extended to understand the response of regional precipitation to GHG and aerosol forcing (10–12). However, the energetic constraints acting on global-mean precipitation are insufficient to explain precipitation changes at the regional scale, where moisture fluxes are controlled by atmospheric circulation. The response of atmospheric circulation is more complex, and its time dependence has only recently started to be explored. Circulation changes can be forced via rapid adjustments, before any sea-surface temperature (SST) warming is realized, due to the direct radiative effects of GHGs on stratospheric (13), tropospheric (14), and land-surface (5) temperatures. However, distinct fast and slow circulation responses to warming also result from the time evolution of SST warming patterns (15, 16). For example, the poleward shift of midlatitude jets in the North Atlantic and Southern Hemisphere (SH) is fully realized in about 10 y from an abrupt increase in CO_2_, despite surface warming taking hundreds of years to fully reach equilibrium (16). The time evolution of the SST warming patterns is a coupled atmosphere–ocean process that is strongly influenced by the interplay between the warming of the mixed layer and of the deep ocean (17–19). The implication is that atmospheric circulation changes cannot be generally assumed to scale linearly with global-mean warming, even after removal of the rapid adjustment component.

The above findings suggest that in regions where atmospheric circulation is important for hydro-climate changes, the standard rapid adjustment plus surface-warming response framework may be inadequate to fully characterize the climate change response. This hypothesis will here be tested by isolating the rapid adjustment and two SST-driven components, one fast and one slow, of the time evolution of the precipitation response to GHG forcing. Considering two SST timescales is itself a simplification, since the patterns in the SSTs are continuously evolving. Nonetheless, the resulting three-timescale framework suffices to provide substantial insights into the time evolution of the hydro-climate response to climate change in Mediterranean-like regions.

## Results

### The Time Evolution of Precipitation in Representative Concentration Pathway 4.5.

**Fig. 1.**
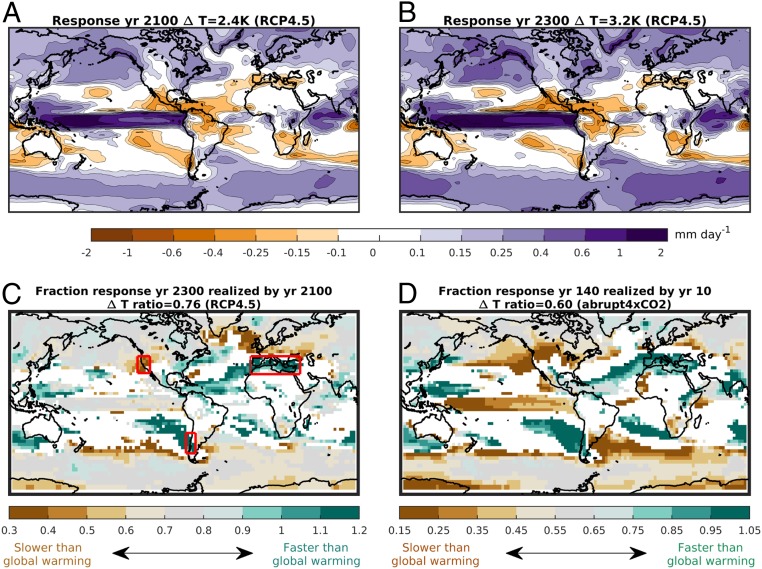
(*A*) CMIP5 multimodel mean annual-mean precipitation response (mm⋅d^−1^) by year 2060 to 2099 in the RCP4.5 scenario relative to 1900 to 1949. (*B*) As in *A*, but for the long-term precipitation response by year 2260 to 2299. In the titles, Δ T indicates the global warming realized in each period. (*C*) Ratio of *A* to *B*, i.e., the fraction of the long-term response already realized by 2060 to 2099. Gray shading implies that the ratio in the precipitation response scales like global-mean warming. Brown (green) shading implies that the precipitation response is slower (faster) than global warming. White shading shows where less than 75% of models agree on the direction of change. (*D*) As in *C*, but for the fraction of the mean response in the abrupt4xCO2 experiment in years 120 to 139 that is already realized by years 5 to 10. The multimodel means are based on all of the CMIP5 models available for each experiment (*SI Appendix*, Table S1). The results in *D* are confirmed both in the subset of seven CMIP5 models that form the basis of this study (*SI Appendix*, Fig. S1*A*) and in an alternative calculation where the rapid adjustment is explicitly accounted for (*SI Appendix*, Fig. S1*B*). The red boxes in *C* show the domains of the area-averaged values in Table 1.

Fig. 1 *A* and *B* present the Coupled Model Intercomparison Project, Phase 5 (CMIP5) multimodel mean transient (2060 to 2099) and long-term (2260 to 2299) annual-mean precipitation changes in the extended Representative Concentration Pathway 4.5 (RCP4.5) emissions scenario (Materials and Methods), relative to the 1900 to 1949 mean climate in the historical runs. In both periods, the precipitation response exhibits well-known features, such as a wetting of the high latitudes and tropical Pacific ocean and a drying of most subtropical regions (20). In this scenario, GHG concentrations are roughly stabilized by 2080 (21), but, due to the thermal inertia of the ocean, mean global warming still increases from 2.4 K in 2060 to 2099 to 3.2 K in 2260 to 2299.

If the projected regional precipitation changes (Δ P) just depended, in a linear way, on global-mean warming (Δ T), we would expect the ratio in the precipitation response between the two periods to be $\Delta \;P_{ratio}=\Delta \;P_{2100}/\Delta \;P_{2300}∼\Delta \;T_{2100}/\Delta \;T_{2300}=0.76$. Conversely, deviations from this value imply that either nonlinearities, rapid adjustments, or the evolution of SST patterns modulate the equilibration of the regional climate responses to GHG forcing. To explore the presence of such behaviors, the ratio in the precipitation response between the two periods ($\Delta \;P_{ratio}$) is presented in Fig. 1*C*.

In several regions, the time evolution of the precipitation response is found to be approximately proportional to global-mean warming (gray shading in Fig. 1*C*; $\Delta \;P_{ratio}∼0.7-0.8$). One such example is the projected wetting of the high latitudes. Here, a linear dependence on surface warming is expected since the high-latitude precipitation increase is fed, through the “wet get wetter” mechanism, via the thermodynamic increase in atmospheric moisture content (22).

A different behavior is found in several subtropical regions (green shading in Fig. 1*C*). Here, most of the long-term precipitation decline is already fully realized within the 21st century ($\Delta \;P_{ratio}∼1$), indicating that the response equilibrates faster than global-mean warming. This behavior was previously noted over the subtropical oceans as a result of circulation changes forced within the rapid adjustment to radiative forcing (5). However, Fig. 1*C* further identifies that subtropical land areas, such as the Mediterranean and parts of Chile, are also characterized by a precipitation decline that evolves faster than global-mean warming. In some areas, $\Delta \;P_{ratio}$ is even larger than unity, indicating that the transient response tends to overshoot the long-term drying.

Interestingly, there are also areas in which $\Delta \;P_{ratio}≲0.5$ (brown shading), implying that precipitation changes equilibrate slower than global-mean warming. In these regions, counterintuitively, most of the climate change response is realized only after GHG concentrations are stabilized. Over land, this behavior is mainly found for the projected increase of precipitation in southwest North America, including California. Indeed, the long-term wetting of the region (Fig. 1*B*) has only weakly emerged by 2060 to 2099 (Fig. 1*A*) in the CMIP5 multimodel mean.

These results indicate that global-mean warming is not always sufficient to determine the future evolution of regional precipitation changes. Over land, the most notable deviations from a linear response to warming are found in three Mediterranean-like climates, particularly the Mediterranean proper, Chile, and California. Given the susceptibility of these regions to future hydro-climate changes, understanding the nature of their nonlinear time evolutions is the focus of the next sections.

### The Three-Timescale Decomposition.

The identified nonlinear precipitation responses to warming in RCP4.5 are also found in response to an abrupt quadrupling of ${CO}_{2}$. This is demonstrated in Fig. 1*D*, which shows the fraction of the multimodel mean precipitation response by years 120 to 139 that is already realized by years 5 to 10 (called the fast timescale in ref. 16) in the abrupt4xCO2 experiment. $\Delta T_{ratio}$ for these two time periods is 0.6. The precipitation response is seen to equilibrate faster than global-mean warming in the Mediterranean and Chile ($\Delta P_{ratio}∼1$), but slower than global-mean warming in southwest North America ($\Delta P_{ratio}∼0.2$). Hence, insights on the precipitation response to GHG forcing in these three Mediterranean-like regions can be directly obtained from the response to an idealized step-like increase in ${CO}_{2}$.

The abrupt4xCO2 response is decomposed into three contributions linked to different timescales (see *Materials and Methods* for details):

• Rapid adjustment: the ultra-fast climate response resulting from the direct effect of ${CO}_{2}$ on atmospheric temperature and land surface, before any warming of SSTs is realized.
• Fast SST-driven response: the climate response mediated by the warming of the ocean surface and sea-ice loss that takes place within the first 10 y of an increase in ${CO}_{2}$.
• Slow SST-driven response: the climate response mediated by the long-term warming of the ocean surface and sea-ice loss following an increase in ${CO}_{2}$.

**Fig. 2.**
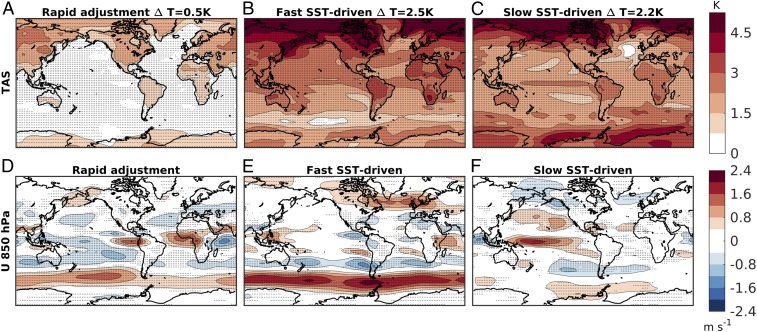
(*A*–*C*) Annual-mean near-surface atmospheric temperature change (K) for the rapid adjustment (*A*) and for the fast (*B*) and slow (*C*) SST-driven responses to quadrupling ${CO}_{2}$. The global-mean surface temperature change (ΔT) is reported in the title. (*D*–*F*) As in *A*–*C*, but for the changes in the zonal wind at 850 hPa. Stippling indicates where at least 85% of the models (six out of seven) agree on the direction of change.

Before discussing the associated precipitation changes, it is informative to first examine the surface warming and circulation responses taking place on these timescales (Fig. 2). By construction, the rapid adjustment only features a warming of the land surface, leading to 0.5 K warming in the global mean (Fig. 2*A*). The fast and slow SST-driven responses (Fig. 2 *B* and *C*) feature a similar amount of global-mean warming (2.5 K and 2.2 K, respectively), which is, however, expressed in different spatial patterns. The fast response shows muted warming in the Southern Ocean and a marked interhemispheric gradient. In contrast, the slow response features muted warming in the North Atlantic and an El Niño-like equatorial warming in the tropical Pacific. These different sea-surface-warming patterns have been shown to force strikingly different responses in the atmospheric circulation (16), here described in terms of the zonal wind at 850 hPa (Fig. 2 *D*–*F*). In particular, the well-known poleward shift of midlatitude westerlies in the North Atlantic and SH is primarily forced by the fast SST response, while the shift of the westerlies in the North Pacific depends on a tug of war between the rapid adjustment and the slow response. These three distinct patterns are robust to the set of analyzed CMIP5 models (*SI Appendix*, Fig. S2). Interestingly, the details in the patterns of SST changes appear to only exert a second-order effect on the fast circulation response, since similar wind changes are also found in response to a uniform warming of the sea surface (*SI Appendix*, Fig. S3). This also suggests that the SST pattern is instead important to explain the missing poleward shift of the jets in the slow response, with implications for the confidence one can place on the climate response on this timescale (Conclusions).

**Fig. 3.**
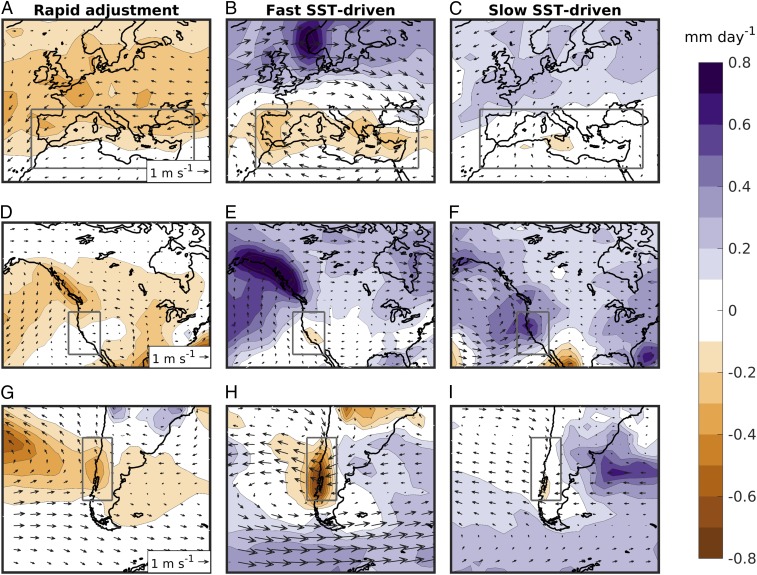
Annual mean precipitation (shading, mm⋅d^−1^) change due to the rapid adjustment (*A*, *D*, and *G*) and due to the fast (*B*, *E*, and *H*) and slow (*C*, *F*, and *I*) SST-driven responses to quadrupling ${CO}_{2}$ in Europe (*A*–*C*), North America (*D*–*F*), and South America (*G*–*I*). The arrows show the direction and speed of the mean wind change at 850 hPa. For clarity, the red boxes from Fig. 1 are also reported here in gray. Information on model agreement on the regional responses can be found in Table 1.

The impact of the different timescales on the annual-mean precipitation change in Europe, North America, and South America is presented in Fig. 3. The patterns and sign of precipitation change vary between the different timescales. The fast SST-driven response contributes to the drying of the Mediterranean and Chile (Fig. 3 *B* and *H*), where it is responsible for 46% and 58% of the annual mean precipitation change, respectively (Table 1). In contrast, the precipitation increase in California is exclusively sustained by the slow SST-driven response (Fig. 3*F*). The rapid adjustment tends to induce precipitation decreases in all three regions (Fig. 3 *A*, *D*, and *G*), so that it enhances the fast drying of the Mediterranean and Chile, while it partially opposes the slow wetting of California.

**Table 1.** The multimodel mean regional precipitation and *P* – *E* responses to quadrupling ${CO}_{2}$ due to the rapid adjustment and to the fast and slow SST-driven components

	Precipitation, mm/d			*P* – *E*, mm/d		
Season	Rapid	Fast	Slow	Rapid	Fast	Slow
Mediterranean						
Annual	**−0.12**	**−0.12**	−0.02	−0.02	**−0.09**	−0.02
Cold	**−0.06**	**−0.18**	−0.00	**−0.02**	**−0.15**	−0.02
Warm	**−0.18**	**−0.07**	**−0.04**	−0.01	−0.04	**−0.02**
California						
Annual	**−0.11**	0.00	**0.34**	−0.02	**−0.06**	**0.22**
Cold	−0.05	−0.08	**0.58**	−0.03	−0.14	**0.47**
Warm	**−0.17**	0.07	**0.11**	−0.01	0.03	−0.02
Chile						
Annual	**−0.23**	**−0.38**	−0.04	**−0.12**	**−0.31**	**−0.11**
Cold	**−0.22**	**−0.47**	0.03	**−0.17**	**−0.41**	−0.04
Warm	**−0.25**	−0.29	−0.11	**−0.07**	**−0.21**	**−0.18**

The responses are evaluated as area averages over land (Materials and Methods) in the three boxes shown in Fig. 3: Mediterranean 29N to 45N, 11W to 40E; California 30N to 45N, 128W to 115W; and Chile 48S to 30S, 78W to 68W. Bold values indicate robust responses (at least six out of seven models agree on the direction of change). In the Northern Hemisphere, cold season is defined as November to April and warm season as May to October, and vice-versa in the SH.

The precipitation reduction due to the rapid adjustment is particularly large in Europe (Fig. 3*A*), and it equals in magnitude the precipitation reduction from the fast response in the Mediterranean (−0.12 mm⋅d^−1^; Table 1). However, we note that the rapid precipitation reduction is always larger in the warm than in the cold season, and these warm-season signals are not reflected in a comparable reduction of precipitation minus evaporation (*P* – *E*) (Table 1 and *SI Appendix*, Fig. S4). This is particularly notable in the Mediterranean (−0.18 mm⋅d^−1^ for precipitation [*P*] against −0.01 mm⋅d^−1^ for *P* – *E*), as well as in California. This implies that the rapid adjustment of precipitation to ${CO}_{2}$ forcing in these three Mediterranean-like land regions is largely mediated by a local suppression of the hydrological cycle, possibly through a stabilization of summer convection. An implication is that the effective impact of the rapid adjustment on water resources is considerably weaker than what would be concluded from inspecting precipitation changes alone.

In contrast to the rapid adjustment, the precipitation responses to SST warming on the different timescales reflect the circulation shifts identified in Fig. 2 *D*–*F*. Indeed, it is immediately notable from Fig. 3 that the different fast and slow SST-driven precipitation responses in the three Mediterranean-like regions are accompanied by strikingly different regional patterns of low-level atmospheric circulation change. In particular, the fast precipitation reduction in the Mediterranean is associated with a localized anticyclonic circulation anomaly, which is absent in the slow response (Fig. 3 *B* and *C*). This anticyclonic anomaly has been previously recognized as a key driver of Mediterranean drying, via enhanced subsidence and a weakening of the regional storm track (1, 23, 24). With regard to California, the slow response features a clear trough anomaly in the Northeast Pacific (Fig. 3 *E* and *F*). Such a trough has been shown to be important in driving an increase in California precipitation via a southward shift of the Pacific storm track and increased moisture advection toward the western coast of North America (25–28). Both the trough and the wetting of California are absent in the fast response, in which the wind-flow anomaly is instead directed toward Alaska. Over South America, the fast poleward shift of the jet is locally manifested as easterly wind anomalies blowing off the coast of Chile (Fig. 3*H*). Similar wind anomalies are also found in the positive phase of the Southern Annular Mode, in which the local weakening and poleward shift of the midlatitude westerlies is known to cause a reduction in Chilean precipitation (1, 29). Finally, in all regions, the SST-driven precipitation changes are largest in the cold season, when they are accompanied by drying or wetting signals of comparable magnitude in *P* – *E* (Table 1). This confirms that the fast and slow precipitation responses to GHG forcing are primarily driven by changes in the horizontal transport of moisture, rather than by local processes. This dependence on atmospheric circulation is confirmed by inspecting the impact of meridional circulation shifts on precipitation in the internal variability of the preindustrial control runs (*SI Appendix*, Fig. S5).

### Interpreting the RCP4.5 Response.

The identified rapid adjustment and the fast and slow SST-driven responses are now used to interpret the time evolution of precipitation under the RCP4.5 stabilized emissions scenario. Any emissions scenario can be considered as the sum of successive small abrupt-like forcings in consecutive years (9, 30). Hence, climate change always comprises fast responses to recently emitted GHGs together with slow responses to previously emitted GHGs, meaning that in a transient emissions scenario, the different timescales of response will overlap, but their relative contributions will vary in time. Here, the contributions of the three timescales in RCP4.5 are estimated via a two-step approach that combines information from both the amplitude of the anthropogenic radiative forcing and the time evolution in the pattern of surface warming (Materials and Methods). The approach assumes that the climate response does not depend on the background state of the climate, that it is linear in the amplitude of the radiative forcing, and that the chosen timescales are sufficient to describe the response to an abrupt increase in GHGs at any lag. Furthermore, influences from other forcers, such as aerosols, ozone, or land-use change, cannot be captured based on the response in the abrupt4xCO2 experiment. Despite these limitations—the climate response is ultimately nonlinear—the approach is seen to provide a remarkable ability to interpret the projected changes in RCP4.5.

**Fig. 4.**
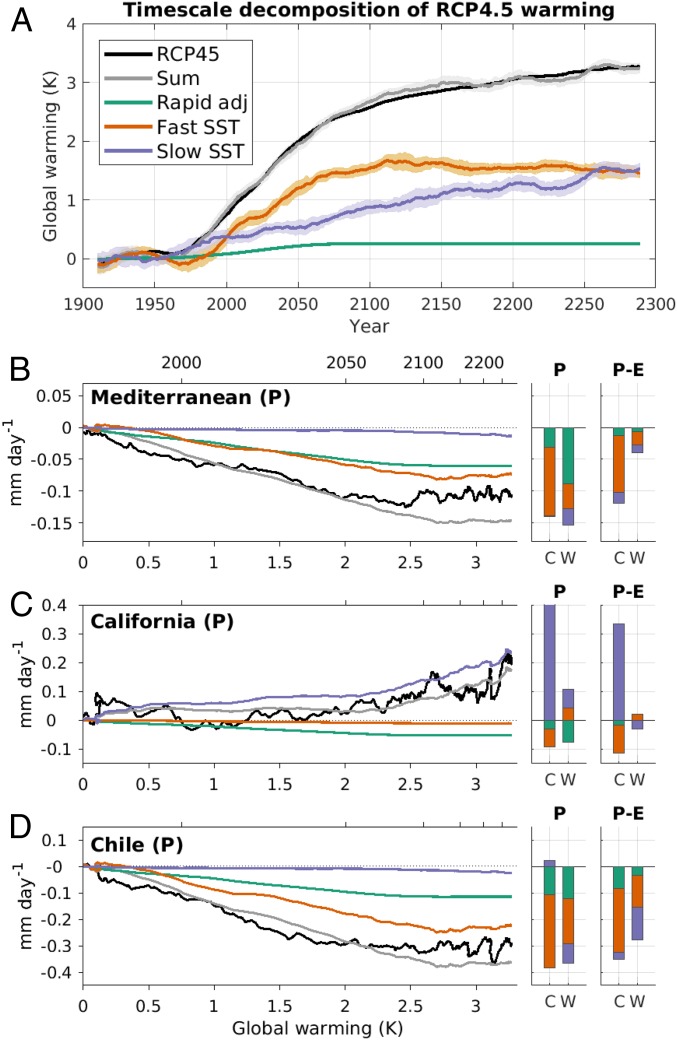
(*A*) Multimodel mean contribution of the rapid adjustment and of the fast and slow SST-driven responses to the global-mean warming in the RCP4.5 scenario. (*B–D*) Decomposition of the annual-mean precipitation changes over land in the Mediterranean (*B*), California (*C*), and Chile (*D*) in RCP4.5 in terms of the three timescales of response. In *B*–*D*, the time evolution of the response is presented against global-mean warming. The equivalent years are indicated on the upper axis. *B*–*D*, *Right* show the timescale contributions to the *P* and *P* – *E* seasonal-mean responses in 2250 to 2299 (C for cold season, W for warm season). These are presented as stacked bars, in which the amplitude of the contribution is given by the length of the bar, separately for the positive and negative values. All time series have been filtered with a 21-y running mean. The shading in *A* represents a 95% CI based on the variability within each 21-y window.

Fig. 4*A* shows the multimodel mean global-mean warming in the RCP4.5 scenario (black line), as well as the estimated contributions to the mean warming from the three timescales of response. Consistent with the evolution of GHG concentrations, the contributions from both the rapid adjustment and the fast components increase until year 2080 and then remain approximately stable. In contrast, the contribution from the slow response keeps increasing until year 2300 and is entirely responsible for the long-term global warming following the stabilization of GHG concentrations in year 2080. The sum of the three components (gray line) well captures the time evolution of global-mean warming projected by the models (black line). There is a slight decrease in the fast component in 2100 to 2300, which would not be expected if the system was entirely linear. However, this is very much a second-order effect, and it does not affect the conclusions of this study.

The evolution of the multimodel mean precipitation response in the Mediterranean, California, and Chile as a function of global-mean warming is presented in Fig. 4 *B*–*D* (black lines). Consistent with Fig. 1, precipitation in the Mediterranean and Chile is projected to decrease until 2050 to 2100 (corresponding to ∼2 to 2.5 K of global warming), after which the signal stabilizes despite ongoing global warming. In contrast, California shows hardly any change in annual-mean precipitation until 2050, but an increasing wetting trend thereafter. These nonlinear time evolutions are found in most individual models. At least six out of the seven models show a change in the trend per degree of warming between 1900 to 2080 and 2081 to 2300 that is in the same direction as the multimodel mean and statistically significant at the 5% level.

The processes responsible for these “climate surprises” with warming can now be understood in terms of the impact of the different timescales of response. This is estimated by multiplying the rapid, fast, and slow precipitation responses (scaled to unit warming) from Table 1 by the corresponding timescale contributions to global-mean warming reported in Fig. 4*A*. The analysis identifies the fast response as the largest contributor to the 21st-century annual-mean precipitation and *P* – *E* reduction in the Mediterranean and Chile (Fig. 4 *B* and *D* and *SI Appendix*, Fig. S7 *A* and *C*). Since the slow response has little impact on these regions, the drying trend saturates shortly after GHG concentrations are stabilized. By contrast, the lack of a climate change signal in California in the 21st century is due to the competing tendencies from the cold-season wetting associated with the slow response and the drying from the rapid adjustment (*P*) and fast response (*P* – *E*) (Fig. 4*C* and *SI Appendix*, Fig. S7*B*). However, the contributions from the latter saturate soon after GHG concentrations stabilize. After that, the slow response wins out, so that annual-mean precipitation increases throughout the 22nd and 23rd centuries.

Considering the strong assumptions underlying the analysis method, the sum of the three contributions captures the projected time evolution of precipitation relatively well, particularly in California. A larger reconstruction bias is found in Mediterranean precipitation, although this is much reduced for *P* – *E* (*SI Appendix*, Fig. S7). The overestimation of the long-term drying in the Mediterranean and Chile is consistent with the presence of nonlinearities in the amplitude of the precipitation response to the magnitude of the radiative forcing in these regions (4). Additional timescales are also likely involved in the response of the Atlantic Meridional Overturning Circulation, which has implications for the long-term evolution of North Atlantic SSTs and, hence, Euro–Mediterranean precipitation (*SI Appendix*, Fig. S8). Finally, as previously mentioned, the effect of aerosols is not explicitly accounted for, which is consistent with the faster-than-predicted drop of Mediterranean precipitation in the late 20th century (31).

## Conclusions

The future hydro-climate response to climate change should not be expected to simply scale with global-mean warming. In the Mediterranean, Chile, and California, the regional hydro-climate response to GHGs is strongly modulated by the time evolution in the SST warming patterns, via the impact it exerts on the midlatitude atmospheric circulation. The rapid adjustment to GHGs also contributes to the precipitation response via a suppression of the local hydrological cycle, but, with the exception of Chile, it plays a negligible role in the response of the water balance (*P* – *E*), which is controlled by circulation changes rather than by energetic constraints.

In the Mediterranean and Chile, the projected drying due to climate change is in quasiequilibrium with GHG forcing, so that the drying trend stops within about a decade of the stabilization of GHGs. This behavior primarily results from the fast SST-driven response, which develops quickly following an increase in GHGs and is particularly effective at inducing a poleward shift of the midlatitude westerlies in both Europe and the SH. These findings revise previous results from He and Soden (2016) (5), who, by examining the rapid adjustment only, concluded that Mediterranean and Chilean drying scales with warming even after GHG concentrations are stabilized. An opposite behavior takes place in California. Here, the projected precipitation increase primarily depends on the circulation change forced by the slow SST response, which takes decades to develop following an increase in GHGs. Hence, the slow wetting of California becomes increasingly apparent after GHG concentrations are stabilized, since, before that, both the rapid adjustment and the fast SST-driven response tend to oppose the wetting of the region.

Note that the rapid adjustment and fast SST-driven responses to GHG forcing are not distinguishable in observations because they are both in quasiequilibrium with smoothly evolving GHG concentrations. However, they reflect different physical processes whose understanding can be addressed through targeted climate-model simulations (16, 17), which is important for gaining confidence in the total fast response. The two components could be physically distinguishable in the case of abrupt forcing, such as from volcanic eruptions. It is thus expected that the three-timescale framework would also be informative in those contexts.

The slow atmospheric circulation response is distinctly different from that to a uniform warming of the SSTs, suggesting that the spatial pattern in the SSTs becomes increasingly important in the slow response. For instance, the trough in the Northeast Pacific and associated California wetting within the slow response are consistent with the slow, El Niño-like SST warming pattern that develops in the Tropics (1). However, due to systematic biases in the representation of tropical climate dynamics, questions remain open on whether the slow, El Niño-like warming pattern is a realistic response to GHG forcing (32, 33). Likewise, the cessation of the drying in the Mediterranean and Chile will also depend on the emergence of the slow SST warming pattern. Understanding the patterns of SST warming that will develop in the real world will be an element of increasing confidence in the future evolution of the regional hydro-climate in these Mediterranean-like regions.

## Materials and Methods

### CMIP5.

The study is based on the analysis of multiple experiments from the CMIP5 (34). The RCP4.5 scenario extended to year 2300 was analyzed to evaluate the climate change response to a gradual increase and stabilization of GHGs. In RCP4.5, GHG concentrations increase to about 580 ppm (${CO}_{2}$-equivalent) in year 2080 and then remain approximately constant until 2300 (21). The climate responses to ${CO}_{2}$ forcing in additional experiments, namely, abrupt4xCO2 and SSTClim4xCO2, were analyzed to understand the RCP4.5 scenario in terms of distinct timescales (see below). To ensure consistency in the analyses, unless where differently stated, the study was based on the subset of seven CMIP5 models that provide all of the experiments needed to estimate the timescales of response as well as the extended RCP4.5 integration to 2300 (*SI Appendix*, Table S1). One ensemble member was analyzed for each model, which is justified given that the focus is placed on understanding robust aspects of the multimodel mean response to climate change.

### Timescales of Response: Definitions.

The climate response to forcing is understood in terms of the contributions from three timescales: the rapid adjustment and the fast and slow SST-driven responses.

The rapid adjustment was evaluated as the difference in the mean climate between the SSTClim4xCO2 and SSTClim experiments. SSTClim consists of 30-y-long atmosphere-only climate model simulations forced by annually repeating SSTs and sea-ice concentrations as obtained from each model’s own climatology in the preindustrial control runs. The same surface boundary conditions, but with quadrupled ${CO}_{2}$ concentrations, were applied in the SSTClim4xCO2 experiment. In such an atmosphere-only setup, the increased ${CO}_{2}$ can only force a climate change response via heating rates in the stratosphere, troposphere, and land surface. Therefore, no SST warming was considered as part of the rapid adjustment. Due to the low thermal inertia of the land, this response can be considered to be rapid and in quasiequilibrium with GHG concentrations.

The SST-driven timescales were estimated from the abrupt4xCO2 experiment, in which coupled climate models were forced by a step-like quadrupling of preindustrial ${CO}_{2}$ concentrations. First, based on ref. 16, the total fast response was defined as the difference in the mean climate in years 5 to 10 of abrupt4xCO2 relative to the preindustrial climatology. From the total fast response, the fast SST-driven component was obtained by subtracting the rapid adjustment defined above. Subtracting the rapid adjustment implies that the fast SST-driven response is ultimately driven by the warming of the ocean surface. The slow SST-driven response was defined as the difference between years 120 to 139 and 5 to 10 in the abrupt4xCO2 experiment. The preindustrial climatology was computed as the average over the first 100 y of the preindustrial control simulation.

### Timescales of Response: Contributions to RCP4.5.

The contribution of the rapid adjustment to the RCP4.5 scenario was estimated by scaling the response to the SSTClim4xCO2 experiment by the total ${CO}_{2}$-equivalent anthropogenic radiative forcing in RCP4.5 (CO2(t)) (21). In particular, the rapid contribution to global-mean warming (R(t)) in year t is given by

$$R\left(t\right)=\left(\frac{\ln\left(CO2\left(t\right)/{CO2}_{ref}\right)}{\ln4}\right)\cdot {\bar{\Delta \;T}}_{rapid},$$ [1]

where ${CO2}_{ref}$ is the average ${CO}_{2}$-equivalent concentration in the reference period (1900 to 1949 in this study), $\Delta \;T_{rapid}$ is the mean surface-warming response to SSTClim4xCO2, and the overbar is a global spatial average.

The contributions of the total fast and slow SST-driven timescales were induced via their distinct patterns of surface warming. In particular, the surface-warming response to the RCP4.5 scenario ($\Delta \;T_{RCP}$) was regressed on the surface-warming patterns (Fig. 2 *A*–*C*) associated with the total fast ($\Delta \;T_{fastTOT}=\Delta \;T_{rapid}+\Delta \;T_{fastSST}$) and slow ($\Delta \;T_{slowSST}$) timescales of response:

$$\Delta \;T_{RCP}\left(t\right)=F\left(t\right)\cdot \frac{\Delta \;T_{fastTOT}}{{\bar{\Delta \;T}}_{fastTOT}}\;+\;S\left(t\right)\cdot \frac{\Delta \;T_{slowSST}}{{\bar{\Delta \;T}}_{slowSST}}+r\left(t\right).$$ [2]

Separately for each year, the coefficients F and S were obtained via a spatial multiple linear regression—weighted by surface area. The coefficients can be interpreted as the contribution of each timescale to global-mean warming in RCP4.5. r is the residual warming pattern not projecting on the two timescales. The contribution of the fast SST-driven response was then obtained by subtraction as F(t)−R(t). The analyses were based on the annual-mean, multimodel mean responses.

The benefit of this two-step decomposition, compared to a simultaneous multiple linear regression on the three timescales of response, is discussed in *SI Appendix* (*SI Appendix*, Fig. S6).

### Spatial Interpolation and Regional Averaging.

For the purposes of presenting spatial maps of multimodel mean fields, the output of each climate model was interpolated to a common T42 horizontal spatial grid, using conservative remapping for *P* and *E* and bilinear interpolation for the other fields. The spatial averages of *P* and *P* – *E* over land were computed on the original uninterpolated model grids, by weighting the field by the fraction of land-surface area in each grid cell.

### Data Availability.

The analysed CMIP5 data are publicly available on the Earth System Grid Federation (https://esgf-node.llnl.gov). The data produced by this study is archived at Figshare, https://doi.org/10.6084/m9.figshare.11764395.

## Supplementary Material

Supplementary File
