# Supplementary material for: Time-evolving sea-surface warming patterns modulate the climate change response of subtropical precipitation over land
Source: Proc Natl Acad Sci U S A. 2020 Feb 18;117(9):4539–45. doi: 10.1073/pnas.1911015117 (PMC7060690; doi:10.1073/pnas.1911015117)
Supplement: Supplementary File [file pnas.1911015117.sapp.pdf]

PNAS
www.pnas.org

Supplementary Information for

Time-evolving sea surface warming patterns modulate the climate change response of subtropical precipitation over land

Giuseppe Zappa, Paulo Ceppi and Theodore G Shepherd

Corresponding Author: Giuseppe Zappa
E-mail: g.zappa@isac.cnr.it

This PDF file includes:

Supplementary text
Figs. S1 to S8
Table S1
References for SI reference citations

www.pnas.org/cgi/doi/10.1073/pnas.1911015117

Supporting Information Text

1. CMIP5 Models

The analyses of this study rely on multiple experiments performed as part of the CMIP5 experimental protocol. Not all modelling groups have run all the experiments needed for the analyses. As indicated in Table S1, 14 models have run the RCP4.5 emissions scenario extended to year 2300, 28 models have run the pre-industrial and abrupt4xCO2 experiments, which are needed to estimate the fast and slow SST-driven responses, and 14 models have run the SSTClim4xCO2 and SSTClim experiments, which are needed to estimate the rapid adjustment. To retain consistency in the analyses, the study is largely based on the subset of 7 CMIP5 models for which the data needed to decompose the RCP4.5 response in the rapid, fast SST- and slow SST-driven responses is available (3-Timescales column in Table S1).

2. Three-timescale decomposition

A. Robustness of the rapid adjustment and of the fast and slow SST-driven responses. Given the limited number of analysed models, we test the robustness of some key results in different sets of CMIP5 models. First, we note that the pattern in the ratio of the transient vs long-term precipitation response in the abrupt4xCO2 experiment in the subset of 7 CMIP5 models (Fig. S1a) is similar to that found in the full set of 28 models that provide the abrupt4xCO2 integration (Fig. 1d in the main paper). Furthermore, we note that the distinct rapid, fast SST- and slow SST-driven responses in the surface warming and in the zonal wind at 850 hPa are also comparable in the mean of the 7 CMIP5 models (Fig. 2) and in the mean of the larger set of 14 CMIP5 models that provide the abrupt4xCO2 and SSTClim4xCO2 experiments (Fig. S2). This suggests that the conclusions of the study are not affected by restricting the analyses to the subset of 7 CMIP5 models that also provide the extended RCP4.5 runs.

Fig. S1b further explores the robustness in the ratio of the transient vs long-term precipitation response in the abrupt4xCO2 experiment, to the removal of the mean rapid adjustment component from both the transient and long-term responses. The rapid adjustment is estimated as the multi-model mean response to SSTClim4xCO2 from all available models. Comparison with Fig. 1d of the main paper reveals changes in the subtropical oceans, where the rapid adjustment is important. However, the overall patterns remain comparable, particularly in the analysed land regions, thus supporting the importance of the evolution in the SSTs.

B. Role of uniform vs patterned SST warming. The atmospheric circulation response to a uniform warming of the SSTs is evaluated as the difference in the climatology of the AMIP4K and AMIP experiments (Fig. S3a), and it is compared to the fast and slow SST-driven responses for the same set of CMIP5 models (Fig. S3b-c). AMIP4K consists of an AMIP experiment in which the SSTs have been uniformly increased by 4K, while sea ice is untouched. Scaled per degree of surface warming, the AMIP4K-AMIP circulation change is similar to that found in the fast response (Fig. S3b, pattern correlation r=0.82). In particular, the zonal wind changes at 850 hPa in the North Atlantic strongly resemble those found in the AMIP4K response. This suggests that the overall warming of the SSTs plays the largest role, although the fast SST warming pattern can still provide a modulating effect. For instance, the stronger response of the westerlies in the SH in the fast response, compared to AMIP4K, is consistent with the enhanced meridional SST gradient in the Southern Ocean. In contrast, there are more marked differences between the slow and AMIP4K circulation responses (r=0.45). This suggests that the details in the SST warming patterns are likely to be important in explaining the lack of a poleward shift of the westerlies in the slow response. For instance, in the Northeast Pacific, the equatorward shift of the westerlies appears to be amplified by the slow SST warming pattern, which is consistent with the tropical El Niño-like SST anomalies. Since sea-ice loss is not included in the AMIP4K perturbation, this approach cannot be used to more formally quantify the role of uniform vs patterned SST warming, which is left for future research.

C. P-E. The main paper highlights how the rapid adjustment exerts a relatively small impact on the P-E response of the Mediterranean, California and Chile, despite being important for the precipitation change. This is further analysed in Fig. S4, which compares the contributions of the three timescales to the multi-model-mean annual-mean P-E response to quadrupling CO_2. For P-E, the rapid adjustment response is smaller than the fast SST-driven and slow SST-driven responses, with the sole exception of the subtropical South Pacific. This supports the view discussed in the main paper that the precipitation reduction forced by the rapid adjustment in the Mediterranean climates is predominantly achieved by a decrease in the intensity of the local hydrological cycle. Fig. S4 further shows that both the fast and the slow P-E responses show a negative change over the Mediterranean sea. This can be expected as a thermodynamic "dry get drier" response to the warmer sea in a region of annual-mean low level divergence. However, the P-E reduction is larger and more markedly extends to the Mediterranean land areas in the fast response, which is consistent with the action of the anticyclonic circulation anomaly (1).

3. Circulation shifts and precipitation changes

The importance of the distinct fast and slow SST-driven circulation changes for regional hydro-climate is tested by using the variability in the pre-industrial control runs as an analog of the future dynamical changes. For simplicity, the circulation changes are evaluated in terms of the latitude of the low-level (850 hPa) westerly jet. In particular, jet latitude is defined by considering the centroid of the meridional distribution of the climatological zonal wind at 850 hPa (2). In this approach, the time-mean zonal wind is first zonally-averaged into a longitudinal sector of interest. Then, the jet latitude is estimated as the average latitude between 20° and 70° weighted by the square of the speed of the westerly flow*. The weighting function is set to zero at latitudes where the mean flow is easterly. To capture the regional circulation shifts relevant for precipitation in the three Mediterranean climates, the following longitudinal sectors are considered: 0E–40E for the Mediterranean, 150W–120W for California, and 90W–60W for Chile.

Fig. S5 shows that, in each region, a poleward positioned jet in the year to year variability tends to be associated with a negative anomaly in the annual-mean precipitation. The difference between the multi-model mean fast and slow responses lies close to the regression line obtained by regressing the precipitation anomalies on the jet latitude anomalies in the year to year variability. This is consistent with the view that most of the difference in the fast and slow precipitation responses in these regions can be attributed to the distinct meridional shifts in atmospheric circulation characterising the fast and slow responses. Interestingly, the individual fast and slow responses tend to lie further from the regression line than their difference in both the Mediterranean and California. This may suggest that thermodynamic factors also play a role but, given the similar amount of surface warming in the fast and slow responses, they tend to cancel out in the fast minus slow difference.

4. Timescale contributions to the RCP4.5 response

A. Robustness of the methodology. In the main paper, the contributions of the three timescales of response to RCP4.5 are estimated by a two-step approach combining information from the magnitude of the anthropogenic radiative forcing and the pattern of surface warming. To evaluate the robustness of this approach, here we explore the extent to which the three timescales' contributions could have been inferred solely based on the pattern of surface warming. We perform a simultaneous multiple linear regression of surface warming in RCP4.5 on the three warming fingerprints associated with the rapid, fast SST-driven and slow SST-driven timescales, i.e. as per Eq. 2 of the main paper but for three rather than two timescales. Hence, the contributions of each timescale to global-mean warming are obtained from the regression coefficients in the multiple linear regression.

Fig. S6 compares results from this alternative method (dashed lines) with those obtained from the two-step approach used in the main paper (full lines). To a first approximation, the two methods give

* 30° to 60° in ref. (2)

comparable time evolutions of the rapid, fast and slow components, thus supporting the robustness of the conclusions of the study. However, the reconstruction based on the simultaneous multiple linear regression tends to overestimate global-mean warming around 2050–2200. This is related to a larger estimate of the fast SST-driven component compared to the two-step approach. As a result, there is a marked non-monotonic time evolution of the fast SST-driven component after 2100, which is not expected in our conceptual framework. The rapid adjustment contribution is slightly reduced, while the estimation of the slow SST-driven component is very similar in the two methods.

The reduced performance of the simultaneous regression suggests that it is difficult to distinguish between the rapid adjustment and fast SST-driven components solely based on their surface warming patterns. There are a number of plausible reasons why this could occur. At root there is the issue that the patterns are not orthogonal, which will increasingly affect the multiple linear regression as more predictors are added to it. Moreover, the regression could be influenced by state-dependencies in the surface warming response, for instance if the impact of the direct effect of CO_2 and of the fast SST warming were not entirely additive on the land surface temperature. Land surface feedbacks are a plausible candidate for such a behaviour. These issues do not affect the separation between the total fast (rapid plus fast SST-driven) and slow components in the two-step approach, which is of most interest in interpreting the difference between the transient and equilibrated climate responses. This supports the choice of using information on the anthropogenic radiative forcing to directly estimate the rapid adjustment in the two-step method.

B. Reconstructions of P-E and surface warming. Fig. S7 shows the contributions of the three timescales of response to P-E area-averaged over land in the Mediterranean, California and Chile. In contrast to precipitation, the rapid adjustment contributes negligibly to the time evolution of P-E in the RCP4.5 scenario. This further supports the notion that the drying of the Mediterranean and Chile are predominantly due to the fast SST-driven response, while the wetting of California is predominantly due to the slow SST-driven response.

Some possible reasons for the presence of reconstruction biases are discussed in the main paper. In this context, Fig. S8 compares the time evolution in the projected and reconstructed surface warming separately computed for the transient (1900 to 2099) and equilibration (2099 to 2299) phases of RCP4.5. The reconstruction based on the rapid, fast and slow timescales tends to overestimate the transient cooling of the North Atlantic ocean, and to underestimate the warming in the equilibration phase. This is presumably due to non-linearities and additional timescales in the response of the North Atlantic overturning circulation to forcing. Such differences in surface warming can be expected to have downstream influences on European and Mediterranean precipitation changes (3), and hence on their reconstruction biases.

Table S1. **List of the CMIP5 models for which model output is available for the experiments analysed in this study. RCP4.5 2300: models providing integrations up to year 2300 forced by the RCP4.5 emissions scenario. abrupt4xCO2: models providing the pre-industrial and abrupt4xCO2 experiments. SSTClim4xCO2: models providing the SSTClim and SSTClim4xCO2 experiments. 3-Timescales: CMIP5 models providing all the main experiments needed for this study. AMIP4K: models providing the AMIP4K and AMIP simulations discussed in the SI.**

Models	RCP4.5 2300	abrupt4xCO2	SSTClim4xCO2	3-Timescales	AMIP4K
ACCESS1-0		x			
ACCESS1-3		x			
bcc-csm1-1	x	x	x	x	x
bcc-csm1-1-m		x			
BNU-ESM		x	x		
CanESM2	x	x	x	x	x
CCSM4	x	x	x	x	x
CESM1-CAM5	x				
CNRM-CM5	x	x			
CSIRO-Mk3-6-0	x	x	x	x	
FGOALS-g2		x			
FGOALS-s2		x	x		
GFDL-CM3		x			
GFDL-ESM2M		x			
GFDL-ESM2G		x			
GISS-E2-H	x	x			
GISS-E2-R	x	x			
HadGEM2-ES	x	x			
inmcm4		x	x		
IPSL-CM5A-LR	x	x	x	x	x
IPSL-CM5A-MR	x	x			
IPSL-CM5B-LR		x			
MIROC5		x	x		x
MIROC-ESM	x	x			
MPI-ESM-LR	x	x	x	x	x
MPI-ESM-MR		x	x		x
MPI-ESM-P		x	x		
MRI-CGCM3		x	x		x
NorESM1-M	x	x	x	x	

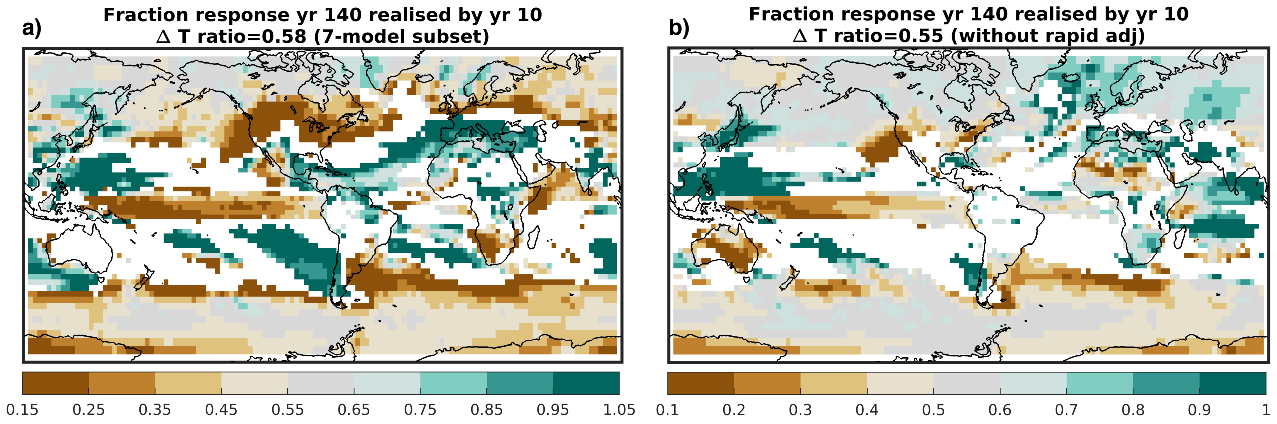

Fig. S1. As in Fig. 1d of the main paper but evaluated a) for the smaller set of 7 CMIP5 models analysed in more detail in the study and b) after the removal of the mean rapid adjustment.

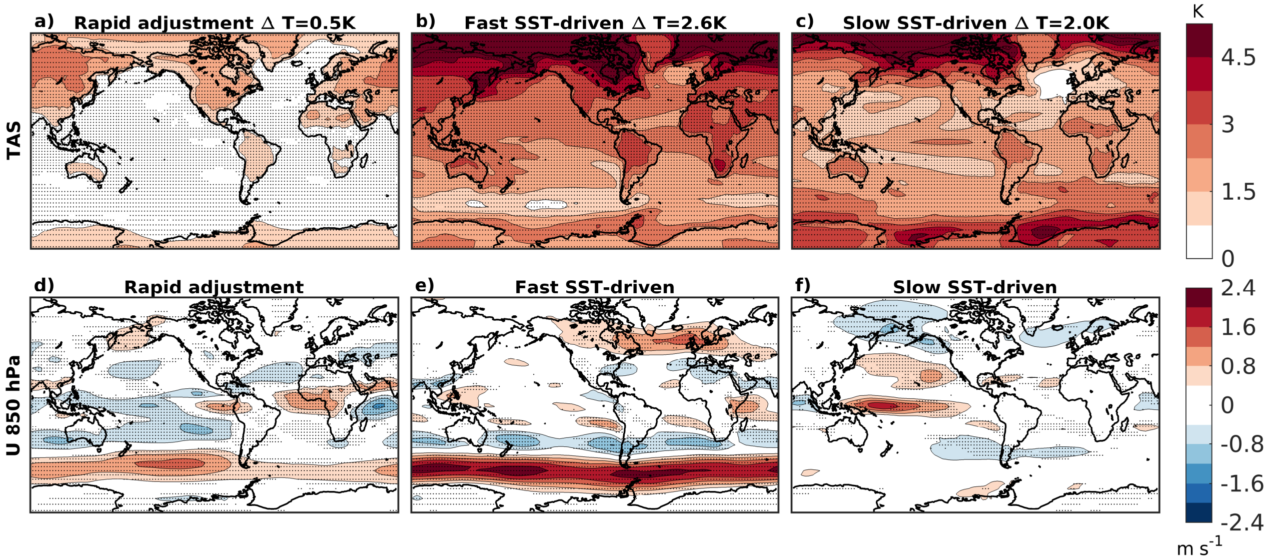

Fig. S2. As in Fig. 2 of the main paper but evaluated for the larger set of 14 CMIP5 models providing the abrupt4xCO2 and SSTClim4xCO2 experiments but not the extended RCP4.5 scenario. The distinct surface warming and circulation patterns identified in the main paper are confirmed in this larger set of models.

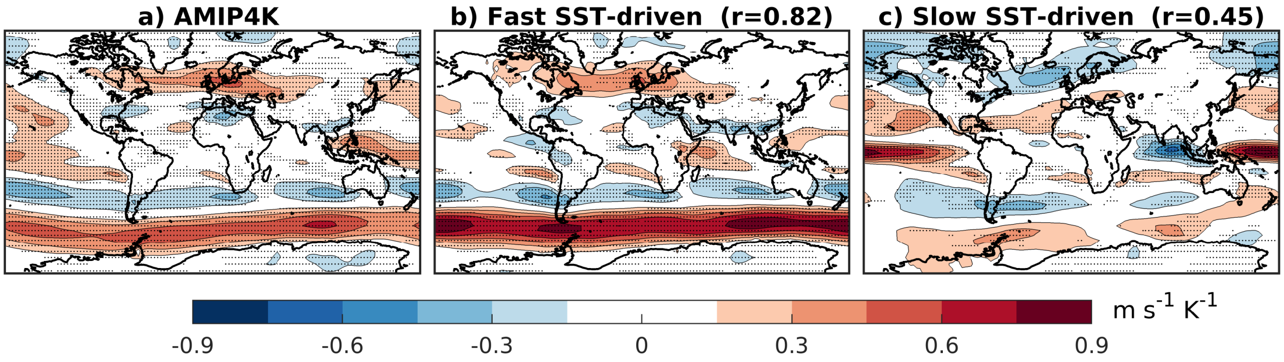

Fig. S3. Comparison of the annual-mean change in the zonal wind at 850 hPa in the a) AMIP4K minus AMIP experiment with the b) fast (SST-driven) and c) slow responses. All responses are scaled per degree of surface warming and evaluated for the same subset of 8 CMIP5 models providing both the AMIP4K and the abrupt4xCO2 experiments (hence panels b) and c) are not identical to Fig. S2e and Fig. S2f, respectively). The pattern correlations, weighted by surface area, between the AMIP4K response and the fast and slow responses are reported in the titles of panels b) and c).

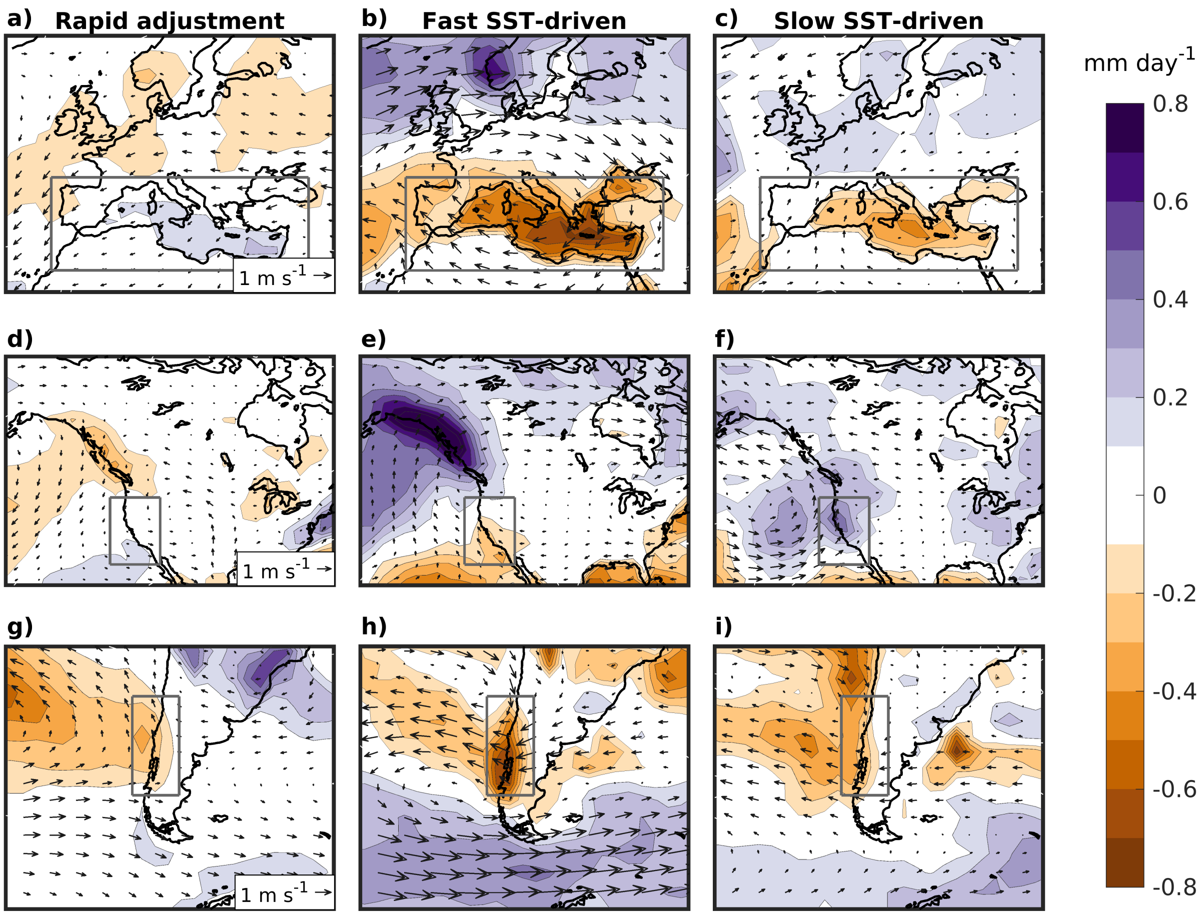

Fig. S4. As in Fig. 3 of the main paper but for the annual-mean P-E response.

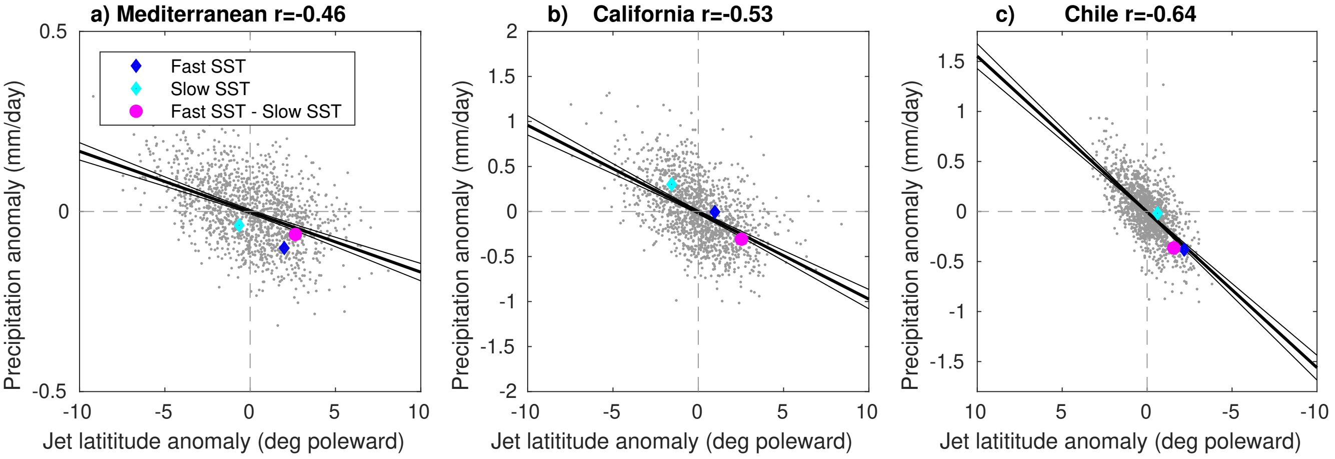

Fig. S5. Relationship between annual-mean regional jet shifts (x-axis) and precipitation anomalies (y-axis) in the Mediterranean (a), California (b) and Chile (c). The coloured dots show the multi-model mean fast, slow and fast minus slow responses. The grey dots show the yearly anomalies in the pre-industrial control runs (years 1–100) for all the CMIP5 models pooled together. The black lines show the linear regression of precipitation on jet latitude anomalies based on the pre-industrial year to year variability, together with the 99% confidence interval on the mean response based on the regression model. The correlation between precipitation and jet anomalies in the year to year variability is reported in the title. This analysis is based on the full set of 14 CMIP5 models providing the SSTClim4xCO2 and abrupt4xCO2 experiments.

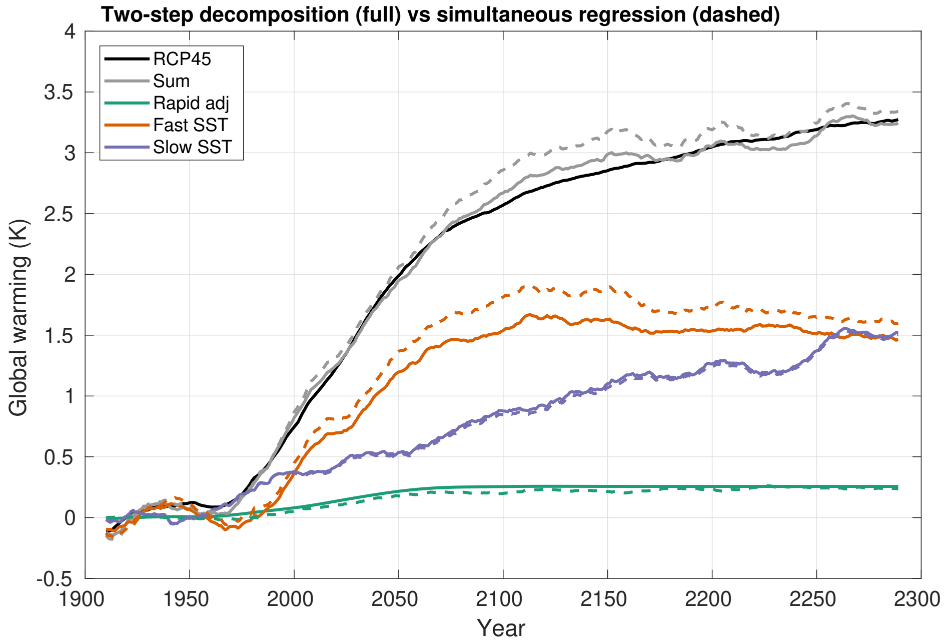

Fig. S6. Comparison of two methods to estimate the contributions of the rapid, fast and slow timescales of response to global-mean warming in RCP4.5. The full lines report the results from the two-step approach, as in the main paper. The dashed lines report the results from a simultaneous multiple linear regression on the three-timescales of response, as discussed in section 4A of this document.

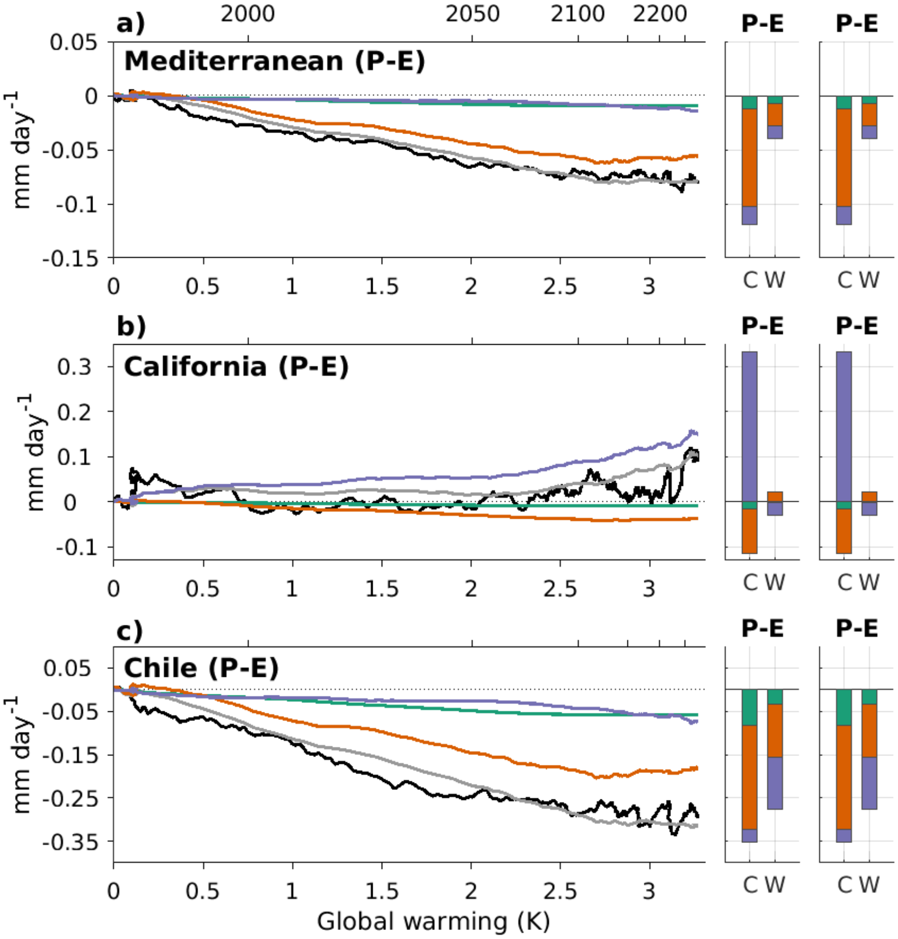

Fig. S7. As in Fig. 4b–d of the main paper, but for the P-E response.

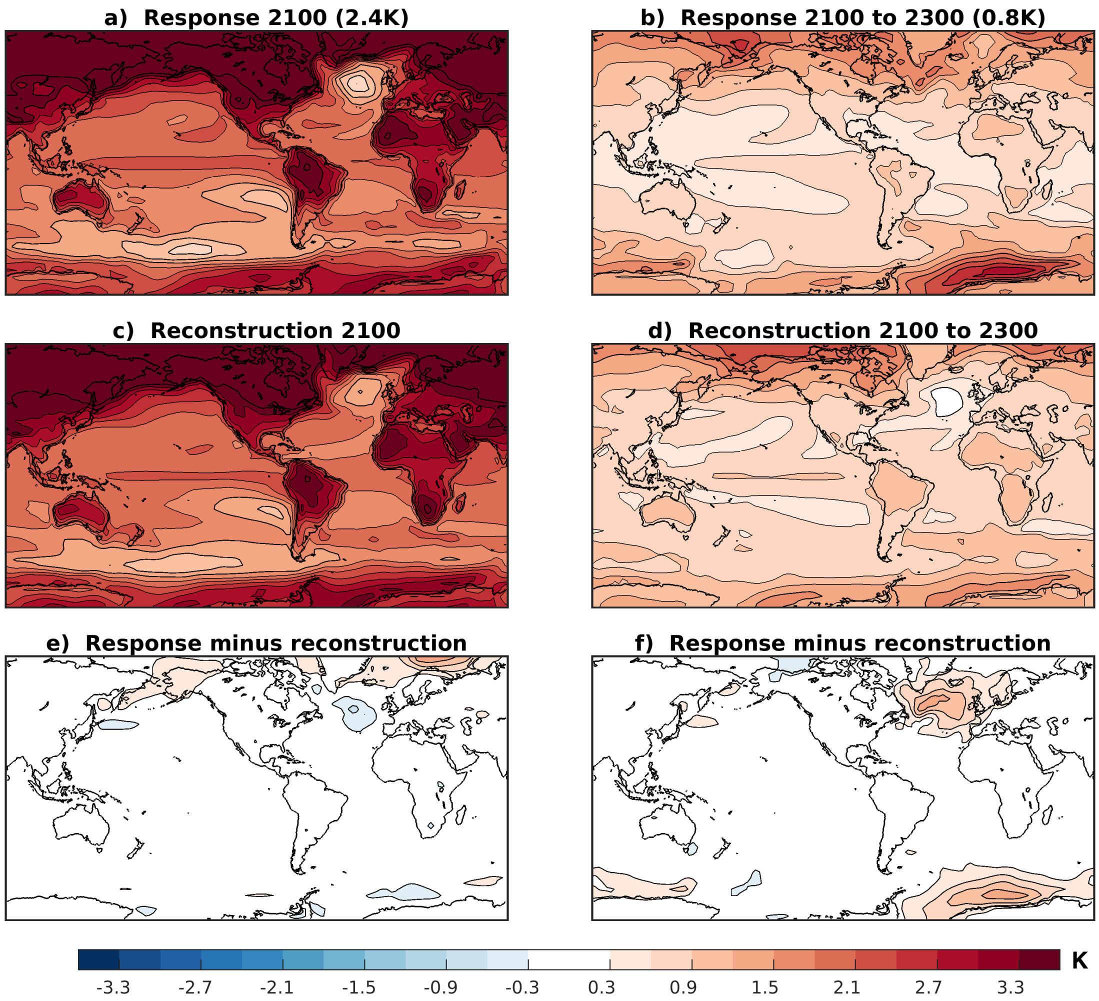

Fig. S8. a) Multi-model mean annual-mean projected changes in near surface temperature (K) in the RCP4.5 transient response (from 1900–1949 to 2060–2099) and b) in the long-term equilibration response (from 2060–2099 to 2260–2299). c-d) as in a-b) but estimated using the three-timescale framework. e–f) Reconstruction biases obtained as panel a) minus c) and as panel b) minus d), respectively.

References

1. Seager R, et al. (2014) Causes of increasing aridification of the Mediterranean region in response to rising greenhouse gases. *J Climate* 27:4655–4676.
2. Ceppi P, Zappa G, Shepherd TG, Gregory JM (2018) Fast and slow components of the extratropical atmospheric circulation response to CO2 forcing. *J Climate* 31:1091–1105.
3. Sutton RT, Dong B (2012) Atlantic Ocean influence on a shift in European climate in the 1990s. *Nat Geosci* 5:788–792.
